# A Cross-National Comparison on Life Expectancy of Non-Hispanic White Americans

**DOI:** 10.3389/ijph.2022.1604603

**Published:** 2022-05-11

**Authors:** Lingling Xie, Defang Xiang, Haijun He, Tiemin Zhai, Zongfu Mao, Xiaohui Liang

**Affiliations:** ^1^ School of Public Health, Wuhan University, Wuhan, China; ^2^ China National Health Development Research Center, Beijing, China

**Keywords:** life expectancy, non-hispanic white, Arriaga method, cause of death, mortality rate, deaths of despair

## Abstract

**Objectives:** Taking the life expectancy (LE) of Non-Hispanic White (NHW) Americans as an example to provide potential references for improving LE globally.

**Methods:** We collected complete data from the United States (US) CDC, Office for National Statistics in the United Kingdom (UK), and the OECD publications, and described LE changes of NHW Americans by cross-national comparison and Arriaga’s method.

**Results:** LE of NHW Americans was not as optimistic as European countries from 2006 to 2018. The LE annual average growth rate was 0.04% for NHW Americans, 0.19% for the UK population, and the median of 25 countries was 0.24%. Compared with the other age groups, the age group 30–34 revealed an inferior impact on the LE of NHW people, of which accidents and intentional self-harm were likely to be the top two direct causes.

**Conclusion:** Finding out the direct causes that affect the LE growth in different age groups is conducive to making a targeted intervention or solving the LE growth bottleneck.

## Introduction

Life expectancy (LE) at birth is an important indicator to assess social health status [1]. China sets a goal of increasing the LE at birth to 79 years old by 2030, and one of Russia’s national development goals is to raise the LE to 80 years old by 2030. However, improving the LE remains a worldwide problem, as the LE growth has slowed in many countries in recent years [2, 3].

Many factors can impact LE, such as advances in public health, modern medical technology [4], health expenditure, genes, and personal behavior. However, from the perspective of an abbreviated lifetable calculation, the mortality rate of each age group is likely to be one of the most significant factors [5]. Therefore, specifically determining the age group that may have the most severe impact on the LE and relevant causes of death would provide references for pragmatic and targeted measures to improve LE.

As one of the developed countries, the total health expenditure in the United States (the US) accounted for 17.7% of GDP in 2018 [6],which was much higher than in other countries. While a pending question is whether such volumed input echoed the country’s health output. Many studies with cross-national comparison methods have demonstrated that the LE of the US population has fallen below other developed European countries [7–9]. Analyzing the reasons behind this phenomenon would be helpful to improve the LE globally.

The U.S. is a multi-ethnic immigrant country, among which, Non-Hispanic White (NHW) people account for 60.5% of the total US population [10]. Therefore, there is no doubt that the LE of NHW Americans would have a great impact on the LE of the US total population. Considering the NHW population has been more comparable to white Europeans in terms of genes and lifestyle, it may be more appropriate to take the Europeans as the comparator in this study.

This study aims to compare the overall LE trend of the NHW Americans with that of the United Kingdom (the UK) and some selected European countries, and to explore the negative influencing factors as well as causes of mortality. The result is expected to provide references for the US and other countries with similar demographic profiles to improve LE in the future.

## Methods

### Data Sources

The LE data of the UK and other European members of the OECD were derived from the OECD official website [11]. There are 25 European countries in the OECD, including Austria, Belgium, Czech Republic, Denmark, Estonia, Finland, France, Germany, Greece, Hungary, Iceland, Ireland, Italy, Latvia, Lithuania, Luxembourg, Netherlands, Norway, Poland, Portugal, Slovak Republic, Slovenia, Spain, Sweden and Switzerland.

The age-specific LE data of the NHW population to be used for Arriaga’s method were collected from the US CDC publications and the National Bureau of Statistics in the United Kingdom [12].

The top five causes of death for NHW in the specific age group were classified by ICD-10 criteria and the information came from the US CDC database [13].

### Cross-National Comparison

Populations from European countries share similar socioeconomic, genetic, cultural, and lifestyle features with NHW Americans. In the meantime, they are also the countries with a predominant proportion of white people. Thus, it is reasonable to do the cross-national comparison to help analyze the LE trend of NHW Americans. Given that quartile values and median are more sensitive to random fluctuations of the LE than the mean value, especially in those countries with relatively small populations, such as Iceland, we particularly examined the first quartile, third quartile, and median value for these countries [2].

### Arriaga’s Method

Arriaga’s method can examine the effect of mortality changes in one age group on LE by controlling the mortality rates in other age groups [14, 15]. This study applied this method to examine the impact of changes in mortality on LE in each age group. The total effect (TE) is an important indicator of the effect of mortality change in one age group on incremental LE. This indicator was measured by a combination of sub-group indicators as follows.

1. Direct effect (DE): years of life gained in the age group x, x + n owing to the mortality changes in the group itself.
2. Indirect effect (IE): years of life gained after the age x + n because of an increased number of survivors (as a result of the mortality changes of the age group x, x + n, assuming the mortality of the older age groups does not change).
3. Interaction: years of life gained because of the supplementary survivors at age x + n as a consequence of mortality changes between x and x + n.

## Results

### NHW Americans Have Lower LE levels Than That of European Countries

In cross-national comparison, the LE of NHW Americans was significantly lower than that of the United Kingdom, and also lower than the median of the 25 European countries. In particular, the LE of females started to become lower than the first quartile of the 25 European countries after 2010, and the LE of males also gradually lagged behind after 2017.

**FIGURE 1:**
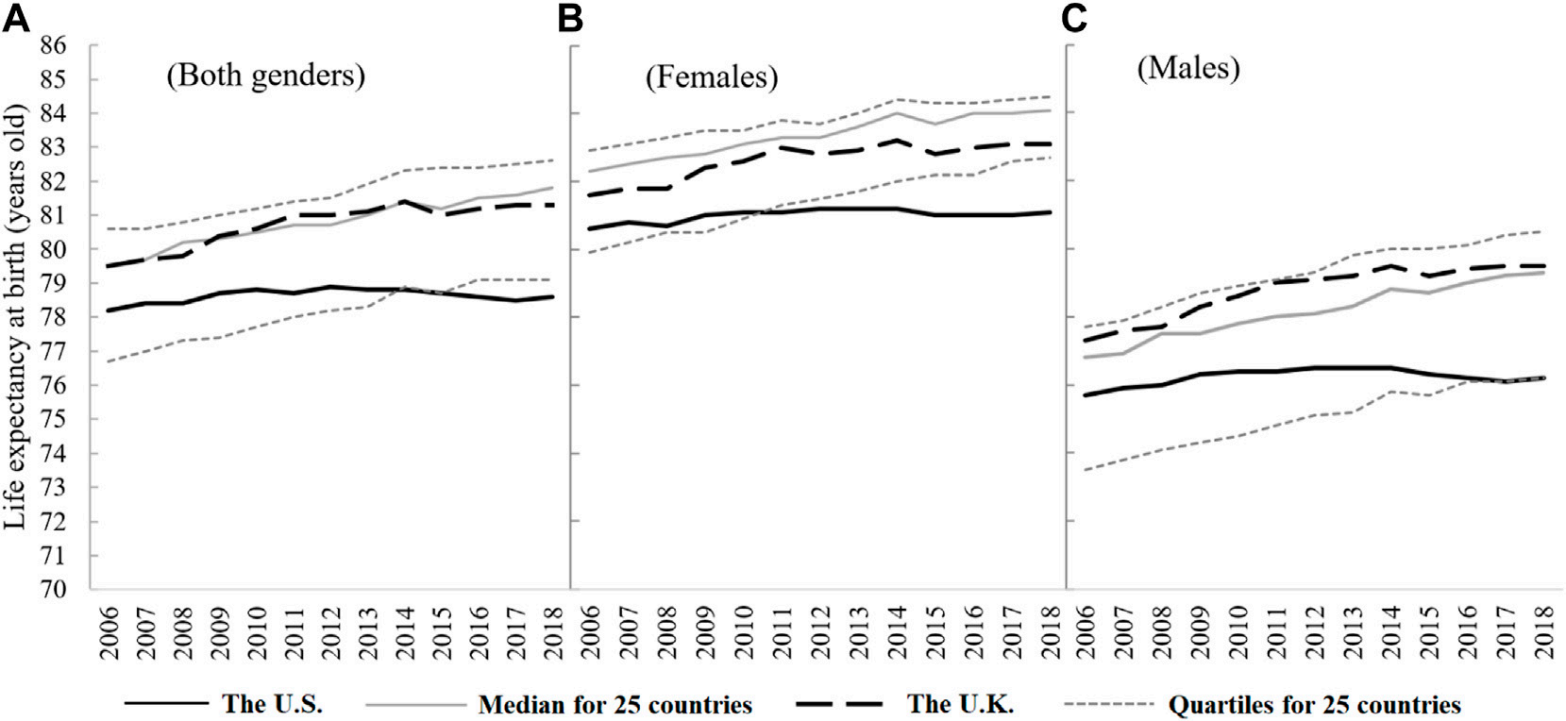
Life expectancy trends of non-hispanic white people in the United States, 2006–2018 (Life expectancy, United States, 2006–2018). **(A)** Both genders. **(B)** Females. **(C)** Males.

Figure 1 shows a significant gap of the LE between NHW Americans and the selected Europeans. The gap has been widening in recent years. From the gender perspective, the gap changes in the LE of males are similar to females but the disparity is wider for males than for females. From 2006 to 2018, the gap between the LE of NHW Americans and the UK widened from 1.3 to 2.7 years. The gap between the NHW Americans and the median of the 25 European countries widened from 1.3 to 3.2 years.

Figure 1 also shows that the speed of LE growth of NHW Americans from 2006 to 2018 was slower than that of other European countries. During the period, the LE of all NHW Americans increased by only 0.4 years, with an average annual increase of 0.04%. Over the same period, the LE of the whole population in the UK increased by 1.8 years, showing an average annual growth rate of more than 4-times higher (0.19%) than that of NHW populations in the United States. The median value of the LE in the 25 European countries increased by 2.3 years, with an average annual increase of 0.24%. The males’ average annual growth rate was higher than that of the females’.

From 2006 to 2012, the LE of NHW in the US rose slowly, that of females increased by 0.6 years and that of males increased by 0.8 years. However, after 2012, the LE of NHW turned down and continued through 2017. In particular, NHW males showed a significant continuous downward trend, from 76.5 years old in 2014 to 76.1 years old in 2017, a decrease of 0.4 years. NHW females experienced a slight setback in 2015, dropping from 81.2 to 81 years old. However, while the median value of the LE in the 25 European countries and the UK also saw some twists and turns in 2015, their LE increased overall during the period. In particular, the median LE of the males in the 25 European countries increased considerably between 2006 and 2018, from 76.8 to 79.3 years old.

### Mortality in the 30–34 Age Group Contributed Most Negatively to the Increment of LE in NHW Americans

In this study, the LE at birth was strongly associated with the mortality in each age group, which could explain the different levels of mortality contribution to LE in different age groups. Therefore, Arriaga’s method was used to identify the age group which had the greatest impact on LE of NHW in the United States.

Table 1 shows the differences in the total effect due to mortality by each age group. If the total effect value is positive, mortality in this age group makes a positive contribution to the increment of LE. The larger the total effect value, the greater the contribution to the increment of LE, and vice versa. From 2006 to 2018, the LE of NHW males in the US increased by 0.477 years, but the mortality of the 30–34 age group led to a 27.4% negative contribution to the increment of LE. In other words, after decomposing the increment of LE by age, the mortality of the 30–34 age group resulted in a decrease of 0.131 years. However, over the same period, the mortality of the British males in this age group contributed positively- an increase of 0.026 years-to the increment of LE.

**TABLE 1:** The mortality effect of each age group on life expectancy for non-hispanic white Americans and the United Kingdom, 2006–2018 (Total effect of life expectancy, United States and United Kingdom, 2006–2018).

	NHW Females in the United States		NHW Males in the United States		Females in the United Kingdom		Males in the United Kingdom	
	TEx	Effects of age group mortality changes on life expectancy (%)	TEx	Effects of age group mortality changes on life expectancy (%)	TEx	Effects of age group mortality changes on life expectancy (%)	TEx	Effects of age group mortality changes on life expectancy (%)
0∼	0.066	13.00%	0.086	18.00%	0.076	5.30%	1.278	57.20%
1∼	0.022	4.40%	−0.001	−0.30%	0.031	2.20%	0.026	1.20%
5∼	0.013	2.70%	0.007	1.40%	0.001	0.00%	0.013	0.60%
10∼	0.005	0.90%	−0.002	−0.40%	0.021	1.40%	0.006	0.30%
15∼	0.026	5.20%	0.047	9.90%	0.011	0.80%	0.037	1.70%
20∼	−0.007	−1.30%	0.05	10.60%	0.012	0.80%	0.056	2.50%
25∼	−0.042	−8.30%	−0.065	−13.70%	0.012	0.80%	0.023	1.00%
30∼	−0.074	−14.70%	−0.131	−27.40%	−0.007	−0.50%	0.026	1.10%
35∼	−0.064	−12.70%	−0.122	−25.60%	0.015	1.00%	0.006	0.30%
40∼	−0.013	−2.70%	−0.019	−4.00%	0.017	1.20%	−0.02	−0.90%
45∼	−0.008	−1.70%	0.026	5.50%	0.021	1.50%	−0.008	−0.40%
50∼	−0.035	−6.80%	0.025	5.20%	0.075	5.30%	0.084	3.80%
55∼	−0.048	−9.60%	−0.064	−13.30%	0.063	4.40%	0.085	3.80%
60∼	0.056	11.10%	−0.012	−2.50%	0.101	7.10%	0.151	6.80%
65∼	0.119	23.50%	0.082	17.20%	0.149	10.40%	0.177	7.90%
70∼	0.148	29.20%	0.155	32.50%	0.224	15.60%	0.201	9.00%
75∼	0.13	25.60%	0.169	35.50%	0.253	17.70%	0.158	7.10%
80∼	0.102	20.20%	0.148	31.10%	0.238	16.60%	0.078	3.50%
85∼	0.088	17.40%	0.083	17.40%	0.094	6.60%	−0.073	−3.30%
90∼	0.025	5.00%	0.016	3.40%	0.025	1.70%	−0.053	−2.40%
95∼	−0.001	−0.20%	−0.001	−0.20%	0.002	0.10%	−0.015	−0.70%
100+	−0.001	−0.30%	−0.001	−0.10%	−0.002	−0.10%	−0.001	0.00%
	0.506	100.00%	0.477	100.00%	1.43	100.00%	2.234	100.00%

The result of the NHW females, breaking down by age group, was very similar to that of males. For females, mortality in the 30–34 age group made the largest negative contribution to the increment of LE than any other age groups, but its negative impact (−14.7%) was smaller than that of males (−27.4%).

### Accidents and Intentional Self-Harm Were to Blame for the Mortality Growth of NHW Americans Aged 30–34 During 2006–2017

**FIGURE 2:**
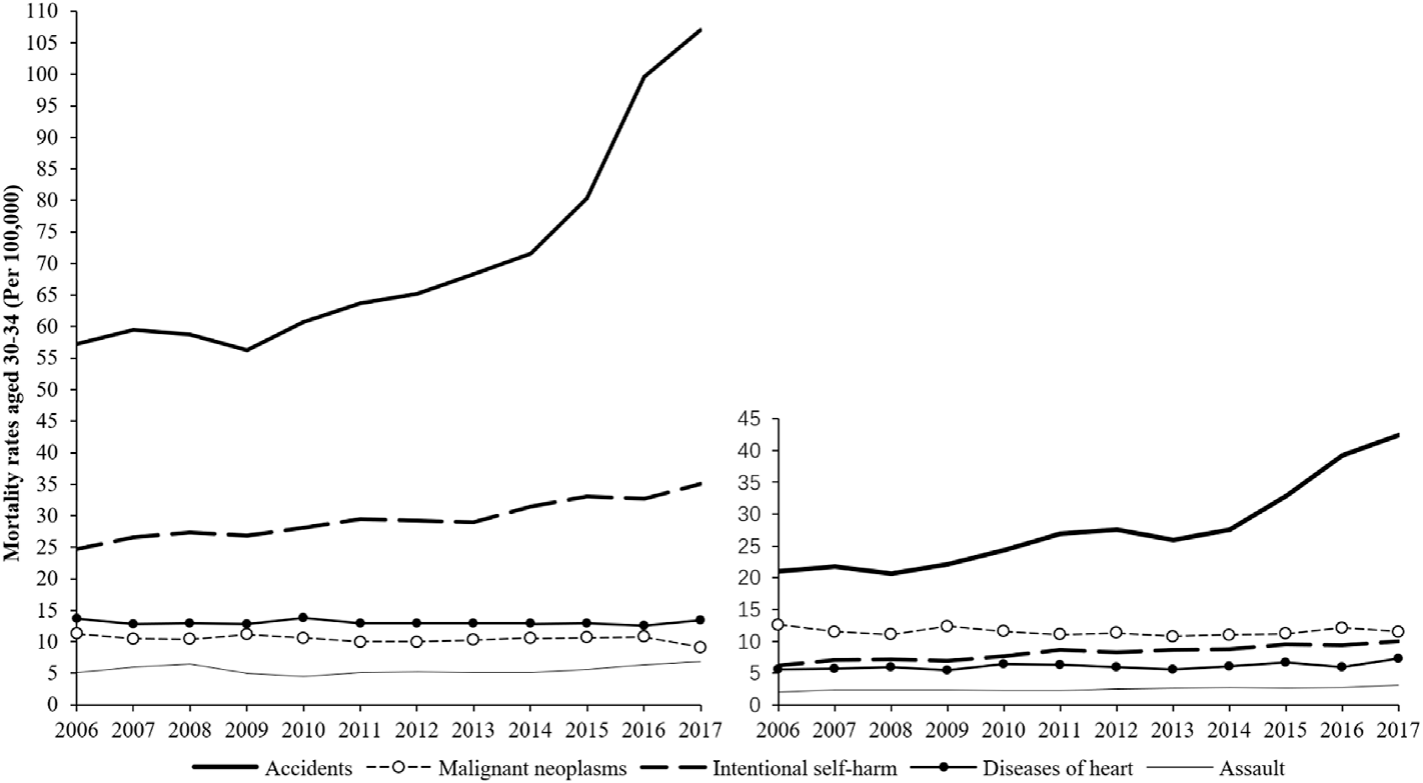
The top five causes of death mortality for non-hispanic white Americans aged 30–34, 2006-2017 (Mortality rate, non-hispanic white Americans, 2006–2017).

Figure 2 shows the top 5 causes of death for NHW Americans aged 30–34 years from 2006 to 2017. They were accidents (unintentional injuries) (V01-X59, Y85-Y86), malignant neoplasms (C00-C97), intentional self-harm (suicide) (U03, X60-X84, Y87.0), heart diseases (I00-I09, I11, I13, I20-I51), and assault (homicide) (U01-U02, X85-Y09, Y87.1). Although the rankings were slightly different between the genders, the fifth cause of death for NHW females in 2016 was chronic liver disease and cirrhosis (K70, K73-K74) (2.8/100,000), instead of assault (2.6/100,000), the rankings within the genders were relatively stable during the period. Males had higher death rates than females in all these causes of death, except for malignant neoplasms. In particular, the mortality of unintentional accidents in males were nearly 3 times higher than that of females.

Accidents were not only the first leading cause of death, but also the fastest growing one. Mortality of accidents increased significantly, regardless of gender. During 2006 to 2017, the annual average death rates grew by 52.5% (from 21.0/100,000 to 42.4/100,000) in females and 53.4% (from 57.3/100,000 to 107.0/100,000) in males. Especially between 2014 and 2017, the death rate of females increased by 14.9/100,000, and that of males increased by 35.5/100,000. According to the US CDC WISQARS (Web-based Injury Statistics Query and Reporting System) database, in 2018, among all types of unintentional injury mortality, poisoning and MV traffic accounted for more than 90% of the death in the NHW Americans aged 30–34 years.

Intentional self-harm was the second top cause of death in males and showed an unstable growth during the 12 years. In 2017, the death rates in males were 35.1/100,000, which was 3.5 times as high as that of females, and 2 times higher than that of the United Kingdom (15.5/100,000). Data from the CDC WISQARS confirmed that the suicide mortality rate in the NHW males was increasing, with 35.9 per 100,000 in the 30–34 age group in 2019, while England and Wales were 16.9 per 100,000 population [16].

The mortality rate of assault ranked fifth in both genders, with relatively slow growth rate. The mortality rates of malignant neoplasms decreased slightly in both genders, and the mortality rate of heart disease was quite stable.

## Discussion

In this study, we found that the LE of NHW Americans was lower than that of some European countries. The result demonstrated that NHW aged 30–34 years had the most negative contribution to the increment of LE. Besides, unintentional accidents and intentional self-harm were the top two causes of death in this age group.

Some currently available cross-national studies revealed similarly sluggish growth in the LE of the Americans [17], but these studies usually did not take the demographic composition into account. In contrast, by following the US Census Bureau’s definition of NHW, the NHW Americans includes persons of European, Middle Eastern and North African origin, we selected European countries in the OECD to control the ethnicity differences and increase comparability. However, our result showed the LE of NHW Americans was lower than that of the UK and the 25 European countries, furthermore, the gap has been increasing. Given the NHW population accounts for the largest proportion of the US total population, the LE trend of NHW can partly explain why the US is lagging behind the European countries.

From 2006 to 2018, per capita health cost in the US increased from $6,821 to $10,624, while it just increased from $3,035 to $4,620 in the United Kingdom [18], but the LE in the US was not as optimistic as that in the UK, and did not rank high in contrast with the European countries. So, it seems that the large volume of health expenditure input of NHW Americans did not echo its health output. However, the LE at birth just one of the health output indicators, it would be irresponsible to judge the US health input-output ratio just by one metric. The output should be evaluated by many aspects [19], for example, the mortality rate of malignant neoplasms for NHW Americans in 30–34 age group was well controlled, which may reveal the clinical medical advances in the United States.

The finding that the 30–34 age group had the most negative influence on the increment of LE concurs with the result done by Kenneth D. Kochanek’s group who pointed out that NHW aged 25–34 contributed a great loss of 0.125 years to the changes in LE from 2000 to 2014 [20]. Besides, our findings on the direct cause of this phenomenon were consistent with those studies on “deaths of despair”—death by drugs, alcohol and suicide, which was brought up by Case and Deaton in 2015 [21]. Actually, both the 45–54 age group, the population studied by Case and Deaton, and the 30–34 age group were also struggling with death of despair.

Two major factors were found to be potentially responsible for the exceptionally outstanding death rate increase of NHW Americans aged 30–34 in the accidents. The first one would be poisoning, which mainly refers to drug overdose. In 2018, there were 67,367 deaths due to a drug overdose in the United States, and NHW accounted for 73.7% (49,637 deaths) of those cases [22]. The overuse of opioids was relatively severe, among all the drug deaths. Additionally, white Americans were more sensitive to opioid deaths than other races, based on the centralized media coverage [23]. The second one is Motor Vehicle (MV) accidents. According to the Association for Safe International Road Travel (ASIRT), the US suffered the most road crash deaths of any high-income country, about 50% higher than Western European countries, Canada, Australia and Japan [24]. This is highly associated with poor driving awareness [25] and growing number of drug users and drunk drivers [26].

Moreover, this study demonstrated that the unstable growth of intentional self-harm or suicidal mortality should not be ignored since the majority of suicides are linked to mental illness. A higher suicide death rate in NHW males than females was probably related to job/financial problems and criminal/legal problems [27]. The number of suicidal deaths among the NHW population in rural areas was higher than that in large or medium-sized metropolitan cities [28]. Therefore, special attention needs to be paid to the rural NHW males aged 30–34 and further analyses on this population’s mental health are necessary.

Age is a factor that is always overlooked in the study of health inequalities and inequities. There have been very few studies which examine health inequities across age groups. Yukiko Asada [29] proposed the idea that health inequality and inequity analysis results vary by choice, as age increases, the ability of individuals to make choices also increases, therefore, this may lead to a reduction in health inequities. Damien Bricard [30] proved that inequalities of opportunity in health and mortality deepen with age, at least until middle age, because of differences in the environment of a person’s upbringing, by a cohort of 17,500 people.

Apart from that, it appears that health inequalities and inequities on different age groups should be based on the background of the era. From 2006 to 2018, all age groups experienced the same development background of the times, especially the same financial crisis and economic recovery. During this period, all age groups of NHW Americans mistakenly believed that their economic and social status was declining, which brought various forms of psychological and physical stress, and finally manifested in the impact on health [31]. But it seems that 30–34-year-olds have more serious health problems than any other age groups because of the bad experiences accumulated by this crisis. This may be because of the social tag on this group—30–34 years old is the decisive point of destiny. A report from 2018 [32] indicated that 30-year-old Americans were exposed to more various types of financial stress, including home loans, car loans, and student loans than any other age groups, which was even higher than their parents’ generation as 30-year-olds. Exposure to so much stress was more likely to lead to mental health problems and risk-taking behaviors [33].

In addition, the total effect calculated by Arriaga’s method is additive. Apart from the group aged 30–34, NHW women aged 20–59 also contribute negatively to LE growth, which indicated that NHW women in the US have an increasing risk of death during their working age [34]. This is consistent with Michelle Anne Parsons’ analysis of NHW mortality in Arizona, which found that all 5-year age groups from 25 to 64 experienced an increase in “deaths of despair”. Both genders have been affected but females had increased by a greater percentage [35]. A long series of negative total effect values, from 20 to 60 years old, demonstrated that the impact of social determinants of health on LE could not be ignored. Social determinants of health and health equity are the most concerned issues in the global health. People’s social status and resources, including income, education, sanitation facilities and living conditions, can have great impact on their health. So, with the age span becoming larger, the influencing factors involved would be much more complicated. It was indicated that corresponding interventions for deaths of despair should be comprehensive, and the measures should be considered to cover a wide range of populations, especially to the working-age ones.

Finding the age group contributing mostly to the loss of LE and the leading causes of death in this age group will help figure out action plans to increase LE and improve the health status of the people. However, complete and continuous data in most developing countries are not available.

In this study, there are three unavoidable data biases: First, since LE data by ethnicity were not available in the United Kingdom, we used the LE of British population included all the ethnicities, among them, British white accounted for about 84% of the total population in 2018. Therefore, we adopted the multi-national median value to enable data comparability. Second, the LE tables for the UK and the US were from corresponding national statistical agencies. Systematic errors may arise due to different sources of the statistical database, nevertheless, the quality of the data was somehow assured as the sources of the data were authoritative. Third, the data on causes of death for NHW in 2018 were unpublished and therefore discarded in this study.

### Limitations

The limitation of this study is that the leading causes of death mortality by age group can only be represented as direct factors of LE, but there are many other social impact factors. Our study did not provide sufficient theoretical discussion about other factors influencing LE but explored the role of age played in the health inequalities and inequities. Besides, we were short of a model to evaluate LE changes by supposing the decline of death rates in each age group. It is expected that future research will focus on analyzing related factors or predicting the increment of LE by modeling a certain decline of death rate in some specific age groups.

### Conclusion

The LE of NHW Americans was not as optimistic as that of the European countries. The age group of 30–34 had the greatest negative influence on the increment of LE for NHW people in the United States. This is largely due to the continuous and significant increase of mortality in accidents and fluctuating growth of mortality in intentional-harm. These findings suggest that we need pay more attention to “deaths of despair” in the 30- to 34-years-old group. The result shows that the discovery and intervention of leading causes of death in special age groups would be helpful to break the bottleneck of LE growth. This result is expected to provide references and clues for other countries to get their LE goals in the future.

## References

[1] Kumari M Mohanty SK. Caste, Religion and Regional Differentials in Life Expectancy at Birth in India: Cross-Sectional Estimates from Recent National Family Health Survey. BMJ Open (2020) 10:35392. 10.1136/bmjopen-2019-035392 PMC7440832 32819936

[2] Leon DA Jdanov DA Shkolnikov VM. Trends in Life Expectancy and Age-specific Mortality in England and Wales, 1970-2016, in Comparison with a Set of 22 High-Income Countries: an Analysis of Vital Statistics Data. Lancet Public Health (2019) 4(11):e575–e582. 10.1016/s2468-2667(19)30177-x 31677776

[3] Santos JV Lobo M Neiva RM Viana J Souza J Dias CC European Union State of Health from 1990 to 2017: Time Trends and its Enlargements' Effects. Int J Public Health (2020) 65(2):175–86. 10.1007/s00038-020-01335-0 32067062

[4] Browne T Keefe RH Ruth BJ Cox H Maramaldi P Rishel C Advancing Social Work Education for Health Impact. Am J Public Health (2017) 107(S3):S229–S235. 10.2105/ajph.2017.304054 29236540 PMC5731074

[5] Murray CJL. The Infant Mortality Rate, Life Expectancy at Birth, and a Linear Index of Mortality as Measures of General Health Status. Int J Epidemiol (1988) 17:122–8. 10.1093/ije/17.1.122 3384530

[6] Hartman M Martin AB Benson J Catlin A. National Health Care Spending in 2018: Growth Driven by Accelerations in Medicare and Private Insurance Spending. Health Aff(2020) 39(1):8–17. 10.1377/hlthaff.2019.01451 31804875

[7] Avendano M Kawachi I. Why Do Americans Have Shorter Life Expectancy and Worse Health Than Do People in Other High-Income Countries? Annu. Rev. Public Health (2014) 35:307–25. 10.1146/annurev-publhealth-032013-182411 24422560 PMC4112220

[8] Woolf SH Aron L. Failing Health of the United States. BMJ (2018) 360:k496. 10.1136/bmj.k496 29437654

[9] Chen A Munnell AH Sanzenbacher GT Zulkarnain A. Why Has US Life Expectancy Fallen below Other Countries? Cent Retire Res A. T Boston Coll (2017) 17:22.

[10] Census Bureau US. About Race (2020). Available from: https://www.census.gov/topics/population/race/about.html. 2020 .

[11] OECD. OECD (1961). Available from: https://stats.oecd.org/# (Accessed January 21, 2022).

[12] Office for National Statistics. Office for National Statistics (2018). Available from: https://www.ons.gov.uk/peoplepopulationandcommunity/birthsdeathsandmarriages/lifeexpectancies/datasets/singleyearlifetablesuk1980to2018 (Accessed January 15, 2022).

[13] NCHS. National Center for Health Statistics (2019). Available from: https://www.cdc.gov/nchs/nvss/mortality_tables.htm#lcod (Accessed January 27, 2022).

[14] Gispert R Serra I Barés MA Puig X Puigdefàbregas A Freitas A. The Impact of Avoidable Mortality on Life Expectancy at Birth in Spain: Changes between Three Periods, from 1987 to 2001. J Epidemiol Community Health (2008) 62:783–9. 10.1136/jech.2007.066027 18701727 PMC2569802

[15] Arriaga EE. Measuring and Explaining the Change in Life Expectancies. Demography (1984) 21:83–96. 10.2307/2061029 6714492

[16] Statistics OFN. Suicides in England and Wales: 2019 Registrations (2019). Available from: https://www.ons.gov.uk/peoplepopulationandcommunity/birthsdeathsandmarriages/deaths/bulletins/suicidesintheunitedkingdom/2019registrations#suicides-in-england-and-wales. 2020 (Accessed February 3, 2022).

[17] Leon DA Jdanov DA Shkolnikov VM. Trends in Life Expectancy and Age-specific Mortality in England and Wales, 1970-2016, in Comparison with a Set of 22 High-Income Countries: an Analysis of Vital Statistics Data. Lancet Public Health (2019) 4(11):e575–e582. 10.1016/s2468-2667(19)30177-x 31677776

[18] Our world in data. Financing Healthcare (2019). Available from: https://ourworldindata.org/financing-healthcare (Accessed February 22, 2022).

[19] Aísa R Clemente J Pueyo F. The Influence of (Public) Health Expenditure on Longevity. Int J Public Health (2014) 59(5):867–75. 10.1007/s00038-014-0574-6 24986366

[20] Kochanek KD Arias E Bastian BA. The Effect of Changes in Selected Age-specific Causes of Death on Non-hispanic White Life Expectancy between 2000 and 2014. NCHS Data Brief (2016) 250:1–8. 10.2105/AJPH.2017.303941 27308863

[21] Case A Deaton A. Rising Morbidity and Mortality in Midlife Among White Non-hispanic Americans in the 21st Century. Proc. Natl. Acad. Sci. U.S.A. (2015) 112(49):15078–83. 10.1073/pnas.1518393112 26575631 PMC4679063

[22] Murphy SL Xu J Kochanek KD Arias E Tejada-Vera B. National Vital Statistics Reports Volume (2018). Available from: https://www.cdc.gov/nchs/products/index.htm (January 12, 2021). 33541516

[23] Gollust SE Haselswerdt J. A Crisis in My Community? Local-Level Awareness of the Opioid Epidemic and Political Consequences. Soc Sci Med (2021) 291:114497. 10.1016/j.socscimed.2021.114497 34710820

[24] ASIRT. Annual Global Road Crash Statistics (2021). AvaliableAt: https://www.asirt.org/safe-travel/road-safety-facts/. 2021 (Accessed March 30, 2022).

[25] National Highway Traffic Safety Administration. Fatal Motor Vehicle Crashes: Overview (2017). AvaliableAt: https://crashstats.nhtsa.dot.gov/Api/Public/ViewPublication/812456. 2017 (Accessed March 27, 2022).

[26] Kahn CA. National Highway Traffic Safety Administration (NHTSA) Notes. Results of the 2013-2014 National Roadside Survey of Alcohol and Drug Use by Drivers. Ann Emerg Med (2015) 66:669. 26918254

[27] Stone DM Holland KM Schiff LB McIntosh WL. Mixed Methods Analysis of Sex Differences in Life Stressors of Middle-Aged Suicides. Am J Prev Med (2016) 51(5):S209–S218. 10.1016/j.amepre.2016.07.021 27745609 PMC7068644

[28] Fitzgerald B Kenzie WR Rasmussen SA Leahy MA Martinroe JC Spriggs SR Morbidity and Mortality Weekly Report Suicide Trends Among and within Urbanization Levels by Sex, Race/Ethnicity, Age Group, and Mechanism of Death-United States, 2001-2015 Centers for Disease Control and Prevention MMWR Editorial and Production Staff. MMWR Editor Board (2017) 66:41. 10.15585/mmwr.ss6618a1

[29] Asada Y Hurley J Grignon M Kirkland S. Health Inequalities and Inequities by Age: Stability for the Health Utilities Index and Divergence for the Frailty Index. SSM - Popul Health (2018) 5:17–32. 10.1016/j.ssmph.2018.04.002 30069499 PMC6066476

[30] Bricard D Jusot F Trannoy A Tubeuf S. Inequality of Opportunities in Health and Death: An Investigation from Birth to Middle Age in Great Britain. Int J Epidemiol (2020) 49(5):1739–48. 10.1093/ije/dyaa130 33011793 PMC7746403

[31] Siddiqi A Sod-Erdene O Hamilton D Cottom TM Darity W. Growing Sense of Social Status Threat and Concomitant Deaths of Despair Among Whites. SSM Popul Health (2019) 9:100449. 10.1016/j.ssmph.2019.100449 31993479 PMC6978487

[32] Kight SW. Being 30 Then and Now Economy & Business (2018). AvaliableAt: https://www.axios.com/one-big-thing-being-30-then-and-now-1531229570-b03dd961-0c1e-4734-a577-78c28ae346d9.html. 2018 (Accessed February 22, 2022).

[33] Seabrook JA Avison WR. Socioeconomic Status and Cumulative Disadvantage Processes across the Life Course: Implications for Health Outcomes. Can Rev Sociology/Revue Can de Sociol (2012) 49(1):50–68. 10.1111/j.1755-618x.2011.01280.x 22586837

[34] Monnat SM. Trends in U.S. Working-Age Non-hispanic White Mortality: Rural-Urban and Within-Rural Differences. Popul Res Policy Rev (2020) 39(5):805–34. 10.1007/s11113-020-09607-6 32921854 PMC7472949

[35] Parsons MA Barger SD. The US Mortality Crisis: An Examination of Non-hispanic White Mortality and Morbidity in Yavapai County, Arizona. J Community Health (2019) 44(4):661–7. 10.1007/s10900-019-00648-3 30877632 PMC7207211

